# The importance of chemosensory clues in Aguaruna tree classification and identification

**DOI:** 10.1186/1746-4269-4-12

**Published:** 2008-05-03

**Authors:** Kevin A Jernigan

**Affiliations:** 1 Ethnobotany program, University of Alaska, Bethel, Alaska, USA

## Abstract

**Background:**

The ethnobotanical literature still contains few detailed descriptions of the sensory criteria people use for judging membership in taxonomic categories. Olfactory criteria in particular have been explored very little. This paper will describe the importance of odor for woody plant taxonomy and identification among the Aguaruna Jívaro of the northern Peruvian Amazon, focusing on the Aguaruna category ***númi ***(trees excluding palms). Aguaruna informants almost always place trees that they consider to have a similar odor together as ***kumpají ***– 'companions,' a metaphor they use to describe trees that they consider to be related.

**Methods:**

The research took place in several Aguaruna communities in the upper Marañón region of the Peruvian Amazon. Structured interview data focus on informant criteria for membership in various folk taxa of trees. Informants were also asked to explain what members of each group of related companions had in common. This paper focuses on odor and taste criteria that came to light during these structured interviews. Botanical voucher specimens were collected, wherever possible.

**Results:**

Of the 182 tree folk genera recorded in this study, 51 (28%) were widely considered to possess a distinctive odor. Thirty nine of those (76%) were said to have odors similar to some other tree, while the other 24% had unique odors. Aguaruna informants very rarely described tree odors in non-botanical terms. Taste was used mostly to describe trees with edible fruits. Trees judged to be related were nearly always in the same botanical family.

**Conclusion:**

The results of this study illustrate that odor of bark, sap, flowers, fruit and leaves are important clues that help the Aguaruna to judge the relatedness of trees found in their local environment. In contrast, taste appears to play a more limited role. The results suggest a more general ethnobotanical hypothesis that could be tested in other cultural settings: people tend to consider plants with similar odors to be related, but say that plants with unique odors are unrelated to any other plants.

## Background

### Chemosensory characters in the ethnobotanical literature

The structure of folk taxonomies of living organisms has been a major focus of the cognitive ethnobotanical literature of the last several decades. A key part of the discussion has been a debate over the relative importance of morphological, ecological and utilitarian factors as bases for folk taxonomies [1-4]. Despite extensive treatment of this subject, the literature still contains surprisingly few detailed descriptions of the specific sensory criteria used for judging membership in taxonomic categories. Some authors [2,5] have included sets of contrasting features for folk species within polytypic folk genera. However, the importance of chemosensory criteria (taste and smell) for folk classification and identification has been addressed very little.

The question of how people distinguish plants with particular kinds of medicinal activities from the many species growing in their local environment is one of great interest to the fields of ethnopharmacology and medical ethnobotany [6-9]. Odor and taste, along with visual and tactile clues tend to be important means for recognizing medicinal plants cross-culturally. In some cases, sensory clues are related to beliefs about illness etiologies. Glenn Shepard [9] reports that the Matsigenka and Yora ethnic groups of the southern Peruvian Amazon believe that foul smelling atmospheric vapors and spirits can cause sickness among people. The Matsigenka use strong smelling aromatic plants to combat these harmful influences. In contrast, the Yora say that the spirits of strong smelling plants can cause illness, and, following a homeopathic logic, are also capable of curing the same illnesses that they cause. Lisa Gollin [6] has investigated the sensory clues that allow the Kenyah Leppo' Ke of Borneo to recognize medicinal plants. Her research particularly focuses on the association of particular kinds of tastes and odor qualities with particular kinds of healing properties. Gollin points out the importance of volatile and essential oils for the medicinal properties of plants. Similarly, Leonti *et al*. [7] report the kinds of odors and, particularly, tastes, that are associated with healing plants among the Popoluca of southern Veracruz, Mexico. Pieroni and Torry [8] have recently compared tastes associated with medicinal activity among Gujarati, Kashmiri and English informants.

Shepard [9] has argued for the need to explore cross-cultural sensory experience from a multidisciplinary perspective. He notes that accounts of sensation from anthropology have often ignored the contributions of the biological sciences. Shepard proposes "sensory ecology" as a new theoretical framework for a cross-cultural understanding of sensation. Sensory ecology would seek to draw both from the scientific understanding of the physiology of sensation and the cultural factors that lead interpretations of sensation to vary within those physiological constraints. Shepard writes "... [S]ensory ecology would be equally interested in cross-cultural variation and similarities and should incorporate physiological understandings and cultural constructions of sensory perceptions within a broad biocultural model addressing human-environment interactions" [[9], p.264].

**Figure 1:**
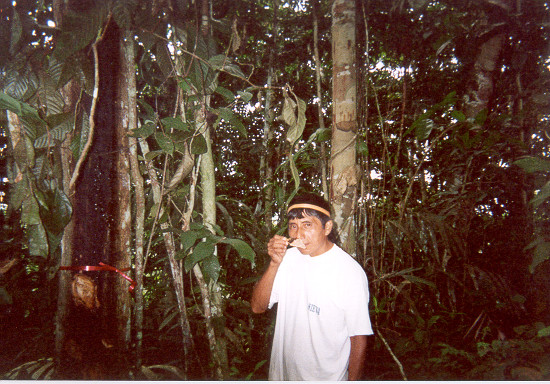
A collaborator smelling the bark of a tree in odor to make an identification.

This paper seeks to clarify the importance of the chemosensory clues odor and taste to classification and identification of trees among the Aguaruna Jívaro of the Peruvian Amazon. It will focus specifically on the Aguaruna folk life-form ***númi ***(tree excluding palms). Bark smelling plays a particularly important role in tree identification among the Aguaruna, since features such as leaves, flowers and fruit can be difficult to examine closely, particularly for large canopy trees (Figure 1). Taste plays a more limited role in identification of woody flora but is still a salient feature of some trees, particularly those with edible fruit. The data that form the basis of this paper were collected during the author's dissertation research [10], which examined sensory and ecological clues that influence the Aguaruna Jívaro of the Peruvian Amazon in their classification and identification of local tree species. A more general treatment of those research results has already been published elsewhere [11]. This article will focus more narrowly on the role that the chemosensory clues taste and, particularly, odor, have for Aguaruna ethnobotany.

### Aguaruna chemosensory vocabulary

In the Aguaruna language, the distinction between smell and taste is not as clear cut as in English. The verb ***kugkúut ***means 'to smell', 'to taste' and also 'to kiss' [12]. Just like the verbs 'to smell' and 'to taste' in English, ***kugkúut ***is used to describe both the production of an odor or taste and the perception of an odor or taste. For example, if an Aguaruna collaborator were to pick a leaf from the shrub ***untuntúp ***(various *Piper *species, Piperaceae) and hold it out for me to smell he might say "***Yatsujú***, ***kugkwásta***" – 'Brother, smell it.' Assuming I could correctly identify the odor, I would respond: "***Yatsujú***, ***untuntúp untuntúp kugkúawai***" – 'Brother, it smells like ***untuntúp***.' The verb ***mejéet ***– 'to reek', in contrast, means specifically to produce an odor, particularly an unpleasant one. Data from this research suggest that the Aguaruna language has very few abstract odor terms. I did encounter the term ***kaujú ***– 'rotten', and the very general ***pégkeg ***– 'good' and ***pégkegchau ***– 'bad.' Other Amazonian languages appear to be richer in general odor terms. Shepard [9], for example lists ten or so abstract odor terms for the Matsigenka and Yora.

The odors of some trees contribute directly to their usefulness. The Aguaruna wear the seeds of ***chikáunia ***(*Myroxylon balsamum*, Fabaceae) and the fruit of ***batút ***(*Ocotea *spp., Lauraceae) on necklaces because of their pleasant fragrance. Only women wear ***chikáunia***. When I asked male Aguaruna informants to describe the odor of this species, they would often answer "***Chikáunia núwa núwa kugkúawai***" '***Chikáunia ***smells like a woman.' On a broader world scale, extracts from *Myroxylon *species have been used in industry for cosmetics [13]. The agreeable odor of the fruit of ***batút ***is considered to bring good luck to ward off illness in general. Rarely, botanical odors can actually be detrimental. One Aguaruna man said of the strong smell of the flowers of ***séetug ***(*Cedrela odorata*, Meliaceae) "***Yagkujín mína bukíeg najámawai***" – 'When it flowers, my head hurts.' The bark, leaves and fruit of this species also have a strong, pungent, almost garlic-like odor.

The Aguaruna verb ***dekapét ***means 'to taste', 'to try' and 'to test' [[12], p.37]. The taste of the fruit, bark or sap are salient characters for some trees. Aguaruna taste terms, include the following: ***yumímitu ***– 'sweet', ***yapáu ***– 'bitter', ***kajiáu ***– 'fermented' ***chujuín ***– 'sour', ***tsupáu ***– 'acrid', ***tajáu ***– 'spicy and ***sákam ***– 'tasteless'. By far the most common taste term used to describe trees is ***yumímitu ***– 'sweet', for edible fruits of many species. Except for ***sákam***, these terms are specific to taste and are never used to describe odors. This fact supports the idea that odor and taste are cognitively separate domains, despite the existence of the verb ***kugkúut***, which encompasses both meanings. Common Aguaruna taste and odor terms are given in Table 1.

**Table 1:** Common Aguaruna odor and taste terms

**ODOR**	
**Term**	**Gloss**

***ámpi***	like medicine
***íki***	like farts
***kaujú***	rotten
***númi***	like a tree
***pégkeg***	good
***pégkegchau***	bad
***perfume***	like perfume
***púku***	like pus
***séj***	like blood

**TASTE**	

**Term**	**Gloss**

***chujuín***	sour
***kajiáu***	fermented
***sákam***	tasteless
***tajáu***	spicy
***tsupáu***	acrid
***yapáu***	bitter
***yumímitu***	sweet

## Methods

**Figure 2:**
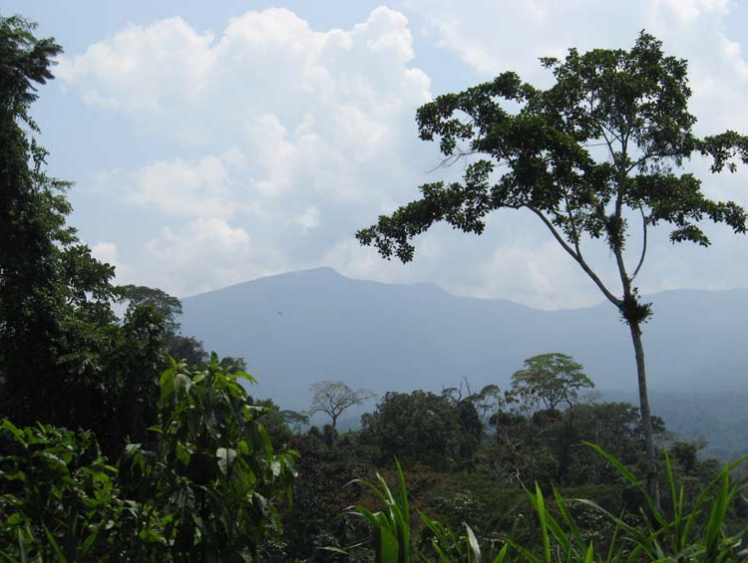
A view of the hill 'Tayuntsa mujaji' from near the study community of Bajo Cachiaco.

**Figure 3:**
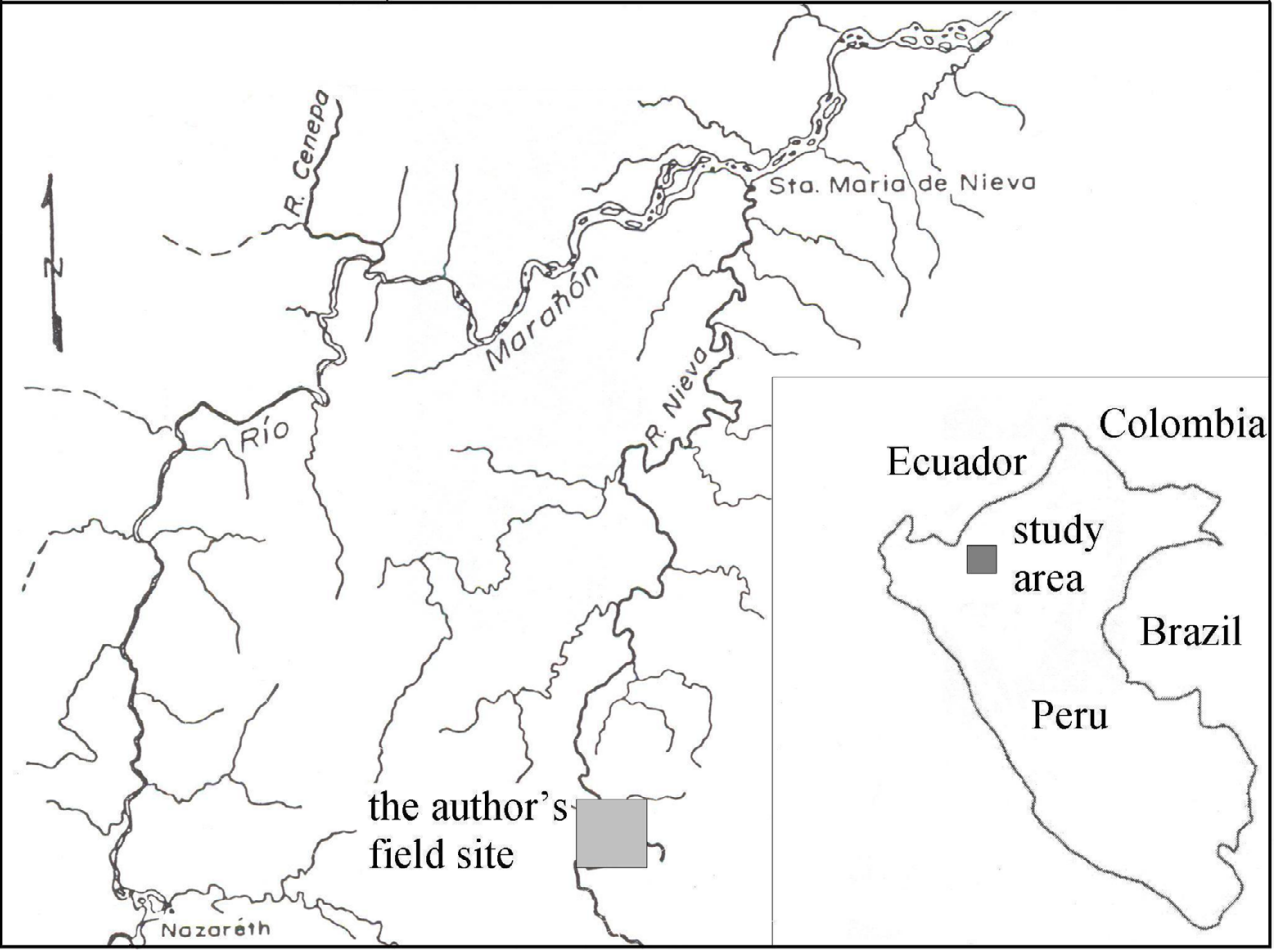
Map of the study area.

The research described in this paper took place in several Aguaruna communities on the Nieva river, from 2002 to 2004 [11] (Figures 2 and 3). Additional follow-up work was also conducted in Summer, 2007. The study communities are located in the eastern foothills of the Andes. Elevations range from 230 m to 500 m above sea level, with mountains up to 1000 m or so in close proximity [[14], pp.42–43]. The communities and adjacent land correspond to tropical wet forest and premontane tropical rainforest in the Holdridge scheme [15].

Fifteen Aguaruna informants participated in interviews designed to determine the most salient morphological and ecological features of folk genera within the Aguaruna life form ***númi ***– 'trees excluding palms.' I obtained verbal prior informed consent (PIC) for every interview. The research followed ethical guidelines adopted by the International Society of Ethnobiology.

For each interview, I first requested the informant to give a freelist of all the trees he could name. These freelist data allowed for the creation of a master list of 182 Aguaruna tree folk genera. Next, I asked informants to list the most prominent features of each tree name (e.g. red sap, large leaves, smooth trunk, grows by the side of streams) in order to get an idea of how each taxon might be recognized and distinguished from other tree taxa [12].

The Aguaruna use the word ***kumpají ***– 'its companion' [11], to describe two or more folk genera that they believe to be related. For example, the trees ***takák ***(*Ocotea gracilis*, Lauraceae) and ***máegnum ***(*Ocotea floribunda*, Lauraceae) are said to be companions, since both have a similar bark odor and fruit shape. As part of the structured interviews, I asked each informant to name any companions of trees mentioned on his freelist. Roughly two thirds of all folk genera were said to have companions, while one third was considered to be unrelated to other trees. Although there was a certain amount of disagreement as to which trees are related, thirty widely recognized groups of companions were found. For each of those groups, informants were asked to explain what members have in common, and how each member is different from others. In twelve of the thirty (40%) of commonly accepted groups, all members shared a common odor. Clearly, odor can be an important clue for recognizing which trees are related.

## Results and discussion

### Structured interviews

For the Aguaruna, many tree odors are idiosyncratic. In other words, people often cannot describe the smells of the bark, sap, fruit or flowers except in terms the tree itself, or other related trees. For example, the likely response to the question: ***Yatsujú, chikáuniash wají wají kugkwáwai***? – 'Brother what does ***chikáunia ***(*Myroxylon balsamum*, Fabaceae) smell like?' would be simply: ***Yatsujú, chikáunia chikáunia kugkúawai ***– 'Brother, it smells like ***chikáunia***.' Clearly, the lack of abstract smell terms creates a problem for cross-cultural understanding. The only way to adequately communicate what ***chikáunia ***smells like to a person who has never smelled it before would be either to cut a piece of bark as an example, or, perhaps, assuming technological feasibility, to extract the essential oils and make a scratch and sniff sticker [10].

Only in a few cases, were informants able to describe the odor of a tree in non-botanical terms. When I asked one informant about the odor of the bark of ***shishím ***(*Couroupita subsessilis*, Lecythidaceae), he replied '***Íki íki mejéawai***' – 'It smells like farts.' Another informant said that the heartwood of the tree ***ugkuyá ***(*Tachigali formicarum*, Fabaceae) smells like ***púku ***– 'pus.' Also, for many informants, the default smell of trees that do not have any other distinctive odor is: '***séj***' – 'blood.' However, other people say of trees with no strong odor simply: '***Númi númi mejéawai***' – 'It smells like a tree.'

Of the 182 tree folk genera recorded in this study, 51 (28%) were widely considered to possess a distinctive odor (see Table 2 in Additional file 1). Informants often said that trees that they considered to be related as companions have similar odors. Thirty-nine of the 51 trees listed in Table 2 (see additional file 1) (76%) have odors that are considered to be similar to some other tree. For example, most people said that the fruits and bark of the trees ***takák ***(*Ocotea gracilis*, Lauraceae) and ***máegnum ***(*Ocotea floribunda*, Lauraceae) have a very similar smell. ***Takák ***and ***máegnum ***are also considered to be companions along with other Lauraceae found in the area. Trees that the Aguaruna consider to have similar odors are almost always in the same botanical family. However, people occasionally compared odors across the Burseraceae and the Meliaceae.

Informants often recognized subtle distinctions among trees they consider to have similar odors. The most important feature uniting members of the Lauraceae in group 4 (see Table 2 in Additional file 1) is the distinctive aromatic odor of the bark and fruit. Gentry also notes that many Lauraceae have a characteristic leaf or bark odor due to the presence of essential oi ls [[16], p.40]. ***Batút ***(*Ocotea *spp., Lauraceae) has an odor that is similar to the other members of its group, but is more rank. ***Káikua ***(*Licaria *sp., Lauraceae) has a particularly rich perfume-like smell.

Aguaruna informants very rarely compared the odors of trees they did not consider to be related. Members of the genus *Protium *and the genus *Dacryodes *(both in the Burseraceae) tend to have a strong odor in the trunk and twig bark, sap and fruit [16]. I find the odor to be similar to frankincense, copal, and also reminiscent of freshly cut dill. The Aguaruna consider members of these two genera to have similar odors, but they do not group them together as companions. One reason most informants separate *Protium *and *Dacryodes *is that the characteristic fruit of each is quite distinct. *Protium *has fruit that dehisce to reveal a soft white aril [[16], p.302] surrounding a single hard seed. *Dacryodes*, however, has indehiscent fruit.

Table 2 (in Additional file 1) contains 12 trees out of 51 (24%) whose odors informants considered to be unique. Trees with very distinctive odors were almost always also said to be unrelated to any other trees as companions. A few informants placed ***tsáik ***(*Cedrelinga cateniformis*, Fabaceae) with ***séetug ***(*Cedrela odorata*, Meliaceae) and ***awán***, which refers to *Cedrela odorata*, or *Swietenia macrophylla *(Meliaceae) [17], because all are important timber trees. Likewise, informants occasionally put the trees ***chíajap ***(various *Trichilia*, Meliaceae), ***ishpíg ***(*Guarea macrophylla *spp. *macrophylla*, Meliaceae) and ***tapákea ***(*Guarea kunthiana*, Meliaceae) with other trees in the Meliaceae or even the Burseraceae. However, none of the above groupings were recognized by a majority of informants.

Some trees have fairly subtle odors. For example, Aguaruna informants typically said that the tree ***magkuák ***(*Cespedesia spathulata*, Ochnaceae) "***imáchik magkuák magkuák kugkúawai***" **- **'smells a little bit like ***magkuák***.' Other trees (see Table 2 in Additional File 1) with odors that are distinctive but weak include ***kántsa ***(various Euphorbiaceae), ***dátash ***(*Aparisthmium cordatum*, Euphorbiaceae), ***káashnum ***(*Eschweilera gigantea*, Lecythidaceae), ***shuwát ***(*Eschweilera *sp., Lecythidaceae), ***chinchák ***(various Melastomataceae), ***ipák ***(*Bixa orellana*, Bixaceae), ***tsáik ***(*Cedrelinga cateniformis*, Fabaceae) and ***tsáchij ***(*Senefeldera inclinata*, Euphorbiaceae). Some informants do not even recognize that the above species have distinctive odors. Interestingly, Gentry [16] also does not mention odor as a clue for recognizing the above taxa.

As mentioned previously, by far the most common taste term that Aguaruna informants used to describe trees is ***yumímitu ***– 'sweet.' I found that this term is commonly applied to just about any fruit that is edible, including ones such as ***apái ***(*Grias peruviana*, Lecythidaceae) that I did not find to be particularly sweet. Of the 182 folk genera collected in this study, 39 (21%) have edible fruit. Informants mentioned taste very rarely for cases other than edible species. One person described the bark of ***wámpa ***(*Inga edulis*, Fabaceae) as ***yumímitu ***– 'sweet.' Some informants described the bark of the trees ***yapúkuit ***(*Ferdinandusa *sp., Rubiaceae) and ***bíchau ***(various Meliaceae) as ***yapáu ***– 'bitter.' The sap of ***tsémpu ***(various Myristicaceae) and the fruit of ***yantsáu ***(various *Guarea*, Meliaceae) were also said to be bitter. Fruit of some trees, such as the cultivated ***toronja ***(*Citrus *sp., Rutaceae) are described as ***chujuín ***– 'sour.' The sap of ***ujúshnum ***(*Croton lechleri*, Euphorbiaceae) is ***tsupáu ***– 'acrid.' I did not encounter any instances of ***tajáu ***– 'spicy' in descriptions of trees, but it does apply quite well for the roots of ***ajég ***(*Zingiber officinale*, Zingiberaceae) and ***ampágpag ***(some *Piper *species, Piperaceae). ***Kajiáu ***– 'fermented' did not appear in any tree descriptions.

## Conclusion

The results of this study illustrate that the odor of bark, sap, flowers, fruit and leaves are important clues that help the Aguaruna to judge the relatedness of trees found in their local environment. In contrast, taste appears to play a minor role. The Aguaruna almost always group trees that have a similar odor as companions. On the other hand, they almost always say that trees with a unique odor have no companions. The utility of odor for judging relatedness is, of course, limited to those trees that have a distinctive odor (slightly less than a third for the Aguaruna). The results suggest a more general ethnobotanical hypothesis that could be tested in other cultural settings. **People tend to consider plants with similar odors to be related, but judge plants with a unique odor to have no relatives.**

In his classic field guide [16], Gentry mentions odor as an important character for recognizing certain plant families, including many that Aguaruna informants also mentioned. For example Gentry observes that trees in the Burseraceae often have an "incenselike or turpentine-like vegetative odor" [[16], p.299] and that trees in the Lauraceae and Myristicaceae typically have a distinctive "Ranalean odor" due to the presence of aromatic essential oils [[16], p.484,638]. Families that both Gentry and Aguaruna informants note for their distinctive odors include: Annonaceae, Burseraceae, Fabaceae (genus *Myroxylon*), Lauraceae, Meliaceae, Monimiaceae, Moraceae (genus *Pourouma*), Myristicaceae, Piperaceae, and Solanaceae. However, the Aguaruna also mentioned some families that Gentry did not. These include the Bixaceae (*Bixa*), Lecythidaceae (genera *Couroupita*, *Eschweilera *and *Grias*), Melastomataceae, Sterculiaceae (*Theobroma*) and Ochnaceae (*Cespedesia*).

Determining the most salient characters of tree taxa for Aguaruna informants is a good first step for approaching the question of how those trees are recognized and identified. While analyzing the data presented in this paper, I had an idea for a follow-up experiment that could shed more light on the question of how the Aguaruna identify trees. The inspiration came partly from Carneiro's [18] classic experiment with the Kuikuru of Brazil, in which he showed a group of men an assortment of leaves he collected from the forest floor in order to elicit identifications of the trees they came from. What follows is a brief outline of the proposed experiment.

The fist step will be selecting a sample of 50 trees representing a variety of families and genera, including trees that informants previously indicated have distinctive odors, such as members of the Annonaceae, Burseraceae, Lauraceae, Meliaceae and Myristicaceae. A few knowledgeable informants will provide an Aguaruna name for each tree in the sample. The second step will involve ten or more key informants. I will cut a piece of bark from each of the 50 study trees and request that each person identify the tree in question, by sniffing it while blindfolded. Thirdly, I will lead each participant through the 50 study trees and request him identify each, using any method he wants. In each case, informants' answers and actions taken while making identifications will be recorded. Finally, I will collect voucher specimens from the fifty trees.

## Competing interests

The author declares that they have no competing interests.

## Supplementary Material

Additional file 1 Table 2 – Trees with distinctive odors. Click here for file
