# Supplementary material for: The importance of chemosensory clues in Aguaruna tree classification and identification
Source: J Ethnobiol Ethnomed. 2008 May 3;4:12. doi: 10.1186/1746-4269-4-12 (PMC2412849; doi:10.1186/1746-4269-4-12)
Supplement: Additional file 1 — Table 2 – Trees with distinctive odors. [file 1746-4269-4-12-S1.pdf]

Additional file 1. Trees with distinctive odors

Aguaruna name	part[i]	odor description	family	species	voucher[ii]
GROUPS OF TREES WITH SIMILAR ODORS					
GROUP 1					
kayayáis	b	yais, 'good'	Annonaceae	*Xylopia parviflora* Spruce	J269
tsáju yáis	b	yais, 'good'	Annonaceae	*Crematosperma* sp.	J263
yúgkua	b	yais, 'good'	Annonaceae	*Rollinia pittieri*	H1080
GROUP 2					
wáwa kúnchai	b,l,fr, s	kunchai, 'good'	Burseraceae	*Dacryodes kukachkana* L.O. Williams	J58
újuts	b,l,fr, s	kunchai, 'good'	Burseraceae	*Dacryodes* sp.	J48
shijíkap	b, l, fr, s	kunchai, 'good'	Burseraceae	*Protium* sp.	J54
chípa	b, l, fr, s	kunchai, 'good'	Burseraceae	*Protium fimbriatum* Swart	J70
pantuí	b, l, fr, s	kunchai, 'good'	Burseraceae	*Protium grandifolium* Engl.	J49
shíshi	b, l, fr, s	kunchai, 'good'	Burseraceae	*Protium grandifolium*	J64
chunchuína			Burseraceae	*Tetragastris* sp.	J69
GROUP 3					
kántsa	b	(slight) kantsa	Euphorbiaceae<br>Euphorbiaceae	*Alchornea glndulosa*<br>*Conceveiba rhytidocarpa*	K1160<br>K322
dátash	b	(slight) kantsa	Euphorbiaceae	*Aparisthmium cordatum*	B937
GROUP 4					
káwa tínchi	b, fr	tinchi, 'good'	Lauraceae	*Nectandra olida* Rohwer	J268
káikua	b, fr	tinchi, 'good'	Lauraceae	*Licaria* sp.	J196
wampúsnum	b, fr	tinchi, 'good'	Lauraceae	cf. *Nectandra schomburgkii* Meisn.	J53
takák	b, fr	tinchi, 'good'	Lauraceae	*Ocotea gracilis* (Meisn.) Mez	J272

batút	b, fr	tinchi, 'good'	Lauraceae<br>Lauraceae	*Ocotea floribunda*<br>*Ocotea* cf. *wachenheimii* Benoist	B875<br>K335
máegnum	b, fr	tinchi, 'good'	Lauraceae	*Ocotea floribunda*	A343
ká̲wa	b, fr	tinchi, 'good'	Lauraceae	*Ocotea floribunda*	A170
GROUP 5					
káashnum	b	(slight) kaashnum, shuwat	Lecythidaceae	*Eschweilera gigantea* (R. Knuth) J.F. MacBr.	J102
shuwát	b	(slight) kaashnum, shuwat	Lecythidaceae	*Eschweilera* sp.	J217
GROUP 6					
antumú chincȟák	b	(slight) chinchak	Melastomataceae	*Miconia* sp.	J149
chijáwe	b	(slight) chinchak	Melastomataceae	*Miconia bulbalina* (Don) Naudin	J112
tseék	b	(slight) chinchak	Melastomataceae	*Miconia ternatifolia* Triana	J75
ukuínmanch	b	(slight) chinchak	Melastomataceae	*Miconia lourteigiana* Wurdack	J267
GROUP 7					
awánu	b, l, fr, fl	seetuj, 'good'	Meliaceae	*Cedrela odorata*	J83
séetug	b, l, fr, fl	seetuj, 'good'	Meliaceae	*Cedrela odorata*	J67
GROUP 8					
yantsáu	b	yantsau, bichau	Meliaceae<br>Meliaceae	*Guarea macrophylla ssp. pendulispica* (C. DC.) T.D. Pennington<br>*Guarea guidonia* (L.) Sleumer	J52<br>K60
bíchau	b	yantsau, bichau	Meliaceae<br>Meliaceae	*Guarea macrophylla ssp. pendulispica*<br>*Trichilia pallida* Sw.	J74<br>KU53
GROUP 9					

kuásip	b, l, fr	'like medicine', 'good'	Monimiaceae	*Siparuna* sp.	J18
tsúna japimágbau	b, l, fr	'like kuasip, but a bit bad'	Monimiaceae<br>Solanaceae	*Siparuna* sp.	J279<br>J280
GROUP 10					
shuíya	stem bark	shuiya	Moraceae	*Pourouma* sp.	J307
tugkápna	stem bark	shuiya	Moraceae	*Pourouma minor*	H693
GROUP 11					
chikúm	b, fr, s	tsempu, 'good'	Myristicaceae	*Virola calophylla* (Spruce) Warb.	J95
ejésh	b, fr, s	tsempu, 'good'	Myristicaceae	*Iryanthera tricornis* Ducke	J80
úntuch tsémpu	b, fr, s	tsempu, 'good'	Myristicaceae	*Iryanthera juruensis* Warb.	J55
takáikit tsémpu	b, fr, s	tsempu, 'good'	Myristicaceae	*Virola* sp.	J135
kadáit tsémpu	b, fr, s	tsempu, 'good'	Myristicaceae		J314
GROUP 12					
untuntúp	b, l, fr	untuntup	Piperaceae	*Piper* sp.	J23
ampágpag	b, l, fr	untuntup	Piperaceae	*Piper* sp.	J311
GROUP 13					
akágnum	b	wakam	Sterculiaceae	*Theobroma subincanum* Martius in Buchner	J184
kúshi wakám	b	wakam	Sterculiaceae	*Herrania* sp.	J308
GROUPS OF TREES WITH UNIQUE ODORS					
ipák	b	(slight) ipak	Bixaceae	*Bixa orellana*	H998

tsáchij	b	(slight) tsachij	Euphorbiaceae	*Senefeldera inclinata* Müll. Arg.	J85, J205
tsáik	b	(slight) tsaik	Fabaceae	*Cedrelinga cateniformis*	J271
chikáunia	b, fr, l	chikaunia, 'good', 'like perfume', 'like a woman'	Fabaceae	*Myroxylon balsamum* (L.) Harms	J207
timúna	b	like timu (*Lonchocarpus utilis*)	Fabaceae	*Pterocarpus amazonum*	H350
ugkuyá	heartwood	'pus', 'blood'	Fabaceae	*Tachigali formicarum*	J264
shishíim	b, fr	shishim, 'bad, 'like farts'	Lecythidaceae	*Couroupita subsessilis* Pilg.	J68
apái	b, fl	apai	Lecythidaceae	*Grias peruviana* Miers	J57
chíajap	b	sometimes compared to other Meliaceae or Burseraceae	Meliaceae	*Trichilia poeppigii* C. DC.<br>*Trichilia septentrionalis* C. DC.	J232<br>J237
ishpíg	b	sometimes compared to other Meliaceae or Burseraceae	Meliaceae	*Guarea macrophylla* spp. *macrophylla*	J226
tapákea	b	sometimes compared to other Meliaceae	Meliaceae	*Guarea kunthiana*	K351
magkuák	b	(slight) magkuak	Ochnaceae	*Cespedesia spathulata* (Ruiz & Pav.) Planch.	J87

[i] b = bark, l =leaves, fr = fruit, fl = flower, s = sap

[ii] Collection numbers preceded by J indicate my own collections, which are deposited in the herbarium of the Universidad Nacional Mayor de San Marcos, in Lima, Peru. Other letters indicate collections from Brent Berlin and his collaborators, as follows: A = Ernesto Ancuash, B = Brent Berlin, H = Victor Huashikat, K = Rubio Kayap, Ku = Kujikat. All material collected by the above collaborators is deposited at the Missouri Botanical Garden, in St. Luis Missouri.
